# The Antioxidant Capacity of Breast Milk and Plasma of Women with or without Gestational Diabetes Mellitus

**DOI:** 10.3390/antiox12040842

**Published:** 2023-03-31

**Authors:** Megan Churchill, Halah Zawawi, Ingrid Elisia, Maxine Seider, Rebecca Noseworthy, Alexandra Thompson, Andrea J. Glenn, D. Dan Ramdath, Deborah O’Connor, Pauline Darling, Thomas Wolever, Douglas E. Barre, Denice S. Feig, David D. Kitts, Shannan M. Grant

**Affiliations:** 1 Department of Applied Human Nutrition, Faculty of Professional Studies, Mount Saint Vincent University, Halifax, NS B3M 2J6, Canada; megan.churchill@msvu.ca; 2 Departments of Obstetrics and Gynecology and Pediatrics, IWK Health, Halifax, NS B3K 6R8, Canada; 3 Department of Nutritional Sciences, Faculty of Medicine, University of Toronto, Toronto, ON M5S 1A8, Canadatwolever@inquis.com (T.W.); 4 The Terry Fox Laboratory, BC Cancer Agency, Vancouver, BC V5Z 1L3, Canada; 5 Department of Clinical Nutrition, Sunnybrook Health Sciences Centre, Toronto, ON M4N 3M5, Canada; 6 Department of Nutrition, Harvard T.H. Chan School of Public Health, Boston, MA 02115, USA; 7 Toronto 3D Knowledge Synthesis and Clinical Trials Unit, Clinical Nutrition and Risk Factor Modification Centre, St. Michael’s Hospital, Toronto, ON M5C 2T2, Canada; 8 Guelph Research and Development Centre, Agriculture and Agri-Food Canada, Guelph, ON N1G 5C9, Canada; 9 School of Nutrition Sciences, Faculty of Health Sciences, University of Ottawa, Ottawa, ON K1N 6N5, Canada; 10 Department of Health Sciences, Cape Breton University, Sydney, NS B1P 6L2, Canada; 11 Department of Medicine, University of Toronto, Leadership Sinai Centre for Diabetes, Mount Sinai Hospital, Toronto, ON M5G 1X5, Canada; 12 Department of Food, Nutrition and Health, Faculty of Land and Food Systems, University of British Columbia, Vancouver, BC V6T 1Z4, Canada; david.kitts@ubc.ca

**Keywords:** infant feeding, gestational diabetes, breast milk, antioxidants, maternal nutrition, high performance liquid chromatography

## Abstract

Women with gestational diabetes (GD) have reduced antioxidant capacity; however, the relationship between maternal diet, maternal biochemical capacity, breast milk concentration, and infant intake has not been adequately explored in the literature. An exploration of underlying mechanism(s) is warranted, particularly for nutrient antioxidants impacted by maternal intake. These nutrients may provide a means for modifying maternal and infant antioxidant capacity. Oxygen radical absorbance capacity (ORAC), alpha-tocopherol, ascorbic acid, and beta-carotene concentrations were measured in breast milk of women with and without GD. Plasma, three-day diet records, and breast milk were collected at 6 to 8 weeks postpartum. Student’s *t*-test was used to compare breast milk ORAC, nutrient antioxidant concentration and plasma ORAC between women with and without GD. Pearson correlations were used to determine associations among antioxidant concentrations in breast milk and dietary antioxidant intake. Breast milk antioxidant concentrations were associated with maternal intake of beta-carotene (*r* = 0.629, *p* = 0.005). Breast milk and plasma ORAC and antioxidant vitamin concentrations were not significantly different between GD and NG women. Breast milk ORAC associated with breast milk alpha-tocopherol for NG (*r* = 0.763, *p* = 0.010), but not GD women (*r* = 0.385, *p* = 0.35), and with breast milk ascorbic acid for GD (*r* = 0.722, *p* = 0.043) but not NG women (*r* = 0.141, *p* = 0.70; interaction *p* = 0.041). In GD participants, breast milk ORAC was significantly associated with plasma ORAC (*r* = 0.780, *p* = 0.039). ORAC and antioxidant vitamin concentrations in breast milk in women with GD were comparable to women with NG; however, the relationships between breast milk ORAC and vitamin concentrations differed in GD versus NG women for alpha-tocopherol and ascorbic acid.

## 1. Introduction

Human milk provides optimal nutrition to support infant growth and development throughout the first six months of life [1,2]. The World Health Organization (2002), Health Canada (2015), the Canadian Paediatric Society (2015), and the Breastfeeding Committee for Canada (2015) recommend that infants be exclusively breast fed/chest fed for the first six months of life, continuing for up to two years and beyond, with the introduction of iron-rich complementary foods at six months [1,2]. Current evidence suggests that breastfeeding can be beneficial for both the mother and the infant, particularly for mothers diagnosed with gestational diabetes (GD) [3]. GD refers to transient maternal dysglycemia during pregnancy and is associated with pre-, peri-, and post-natal complications. During late pregnancy, women with GD and their infants experience increased oxidative stress (OS) compared to those without GD [4,5,6,7]. Although normal human pregnancy is considered a “diabetogenic state”, characterized by increased OS due to the increase in metabolic activity, GD has been associated with an even greater state of OS [4,8,9].

OS results from an imbalance between the production of reactive oxygen species (ROS) and the human body’s antioxidant defenses. ROS are molecules containing oxygen that either have one or more unpaired electrons, or are easily oxidized to form free radicals, which have been associated with molecule and tissue damage in the human body, and are associated with several chronic diseases (e.g., nephropathy, cancer) [9]. Several proxy measurements exist for the direct measurement of ROS, including nutrient and non-nutrient biochemical tests [10,11,12,13]. Nutrient antioxidants found in breast milk include alpha-tocopherol and beta-carotene, both of which are lipophilic vitamins, and ascorbic acid, a hydrophilic vitamin. Each of these antioxidants have also been measured in plasma and are potentially impacted by dietary intake. Lactoferrin (functional protein) and glutathione (nitrogen containing non-protein compound synthesized from glycine, cysteine, and glutamate), as well as antioxidant enzymes such as catalase, superoxide dismutase, and glutathione peroxidase, can also be measured in breast milk to evaluate antioxidant capacity [14,15,16]. Antioxidants in breast milk have been identified as possible and likely systemic defense mechanisms against OS reactions in infants, particularly in their gastrointestinal tract [17,18,19,20,21]. Moreover, research has demonstrated that the addition of antioxidants to infant formula increases infant resistance to OS [19,22].

Several studies that have compared biochemical assays for antioxidant and oxidant quantification unanimously agree that one assay alone does not provide complete antioxidant capacity. Therefore, it is common practice to apply several assays to quantify antioxidant capacity. Total antioxidant capacity (TAC) provides a general assessment of the antioxidant capacity of a given bioactive component, while the quantification of nutrient antioxidants, specific antioxidant enzymes, conjugated dienes, or lipoprotein oxidation provides more specific information [23,24,25]. TAC may be measured by oxygen radical absorbance capacity (ORAC), a method that measures the antioxidant capacity of a substance [26]. The ORAC assay measures a fluorescent signal from a probe that is quenched in the presence of ROS. Modifications of the ORAC assay include the use of fluorescein as the fluorescent probe for the separation of hydrophilic and lipophilic antioxidants, such as ascorbic acid and alpha-tocopherol, to obtain total antioxidant capacity [26].

Women with GD are prone to OS, but there are a limited number of works in the literature assessing this condition from (1) the assessment of breast milk as an available source for infant intake, (2) the assessment of antioxidant composition in maternal plasma, and (3) the examination of maternal intake of dietary sourced antioxidant. We hypothesized that women diagnosed with GD would produce breast milk with decreased antioxidant capacity and have lower plasma ORAC than normoglycemic (NG) women. The objective of this study was to determine maternal breast milk and plasma antioxidant capacity, respectively, between GD and NG groups. This research was conducted in preparation for an analysis of primary and secondary outcomes from a multi-site randomized control trial (ClinicalTrials.gov Identifier: NCT01589757, Short title: GI in GD Study) [27,28].

## 2. Materials and Methods

### 2.1. Study Design

We compared study outcomes in a group of recently pregnant women who were diagnosed with GD to a group of recently pregnant women unaffected by clinical dysglycemia (normoglycemia, NG). Women diagnosed with GD were enrolled in a main prospective multi-centre randomized controlled trial, which aimed to explore the effectiveness of a low glycemic index diet in GD treatment. A matched NG sample was recruited to collect breast milk for comparison to GD participants at three study sites. This research received ethics approval from the University of Toronto, Office of Research Ethics, St. Michael’s Hospital, Sunnybrook Health Sciences Centre, and Mount Sinai Hospital (Toronto, ON, Canada). Approval for secondary data analysis and manuscript writing was obtained from Mount Saint Vincent University, Research Ethics Board (Halifax, NS, Canada).

### 2.2. Sample

Participants were recruited from two studies from October 2011 to November 2011 and January 2013 to May 2014. The inclusion criteria for the main prospective multi-centre randomized controlled trial study were: (1) ≥ 18 years of age, (2) being followed within one of the three pre-selected clinics (St. Michael’s Hospital, Sunnybrook Health Sciences Centre, or Mount Sinai Hospital), (3) willing and able to give informed consent, (4) willing and able to comply with the study protocol. The exclusion criteria for the main prospective multi-centre randomized controlled trial study were: (1) women with acute or chronic illness or use of medications (other than insulin) which may affect carbohydrate metabolism, gastrointestinal function, or carbohydrate digestion (e.g., Crohn’s disease, liver disease, kidney disease, etc.), (2) women known to have type 1 or type 2 DM prior to pregnancy, (3) women known to have multi-fetal pregnancy at enrolment, (4) ≥33 weeks gestation, (5) prescribed oral anti-hyperglycaemic medication, and (6) insurmountable language barriers. Women with GD were invited to participate in this research if they were diagnosed with GD or impaired glucose tolerance of pregnancy according to Diabetes Canada Clinical Practice Guidelines (2018) at 36 weeks gestation [27,29].

### 2.3. Study Objectives and Outcomes

The objective of this project was to compare the antioxidant capacity between women with and without GD following their most recent pregnancy. The study outcomes were: (1) breast milk, (2) maternal plasma, and (3) maternal dietary intake. During study visits one and two (29 weeks gestation and 35 weeks gestation, respectively), participants in the GD group provided blood samples and all participants provided a 3-day diet record. During study visit 3 (8 weeks postpartum), participants in the GD group provided blood samples, breast milk samples, and a 3-day food record; women in the NG group provided breast milk samples and a 3-day diet record.

### 2.4. Breast Milk Sampling and Analysis

Participants provided a complete breast milk expression from the right breast, at 6 to 8 weeks postpartum, between 08:00 and 12:00 using an electric breast pump. Individual breast milk samples were collected from subjects into sample bottles wrapped in aluminum foil to prevent light exposure [30]. Samples were inverted to produce a homogenous mixture, and a total of 25 mL of breast milk was pipetted into 1 mL and 2 mL amber microtubes and immediately placed on dry ice. Microtubes were then stored at −80 °C and shipped on dry ice by World Courier of Canada Ltd. (HZ, TW, SG) (from Toronto, ON to Vancouver, BC, Canada). Breast milk was immediately stored at −80 °C and analyzed in the Department of Food Science, Nutrition and Health, University of British Columbia.

Breast milk TAC was estimated using ORAC, a standardized and validated method to measure the antioxidant capacity in biological samples in vitro [25]. A phosphate buffer was prepared by mixing 0.75 M K_2_HPO_4_: 0.75 M NaH_2_PO_4_ to 61:39 (*v*/*v*); the phosphate-buffered solution was diluted to a final concentration of 75 mM and pH = 7. Fluorescein sodium salt was diluted to a final concentration of 200 nM, and peroxyl radical initiator 2,2-azobis (2-amidinopropane) dihydrochloride was dissolved to a final concentration of 60 mM immediately prior to use. Lastly, concentrations of Trolox (a water-soluble vitamin E analogue) standard (0.0–4.0 mM) were prepared immediately prior to use. Calculations were performed according to Davalos et al. and Ou et al. [31,32]. The final ORAC values for breast milk samples were expressed as an equivalent of the μmol concentration of Trolox standard per milliliter of breast milk (mmol TE/mL milk). All analyses were performed in triplicate.

Ascorbic acid presence in breast milk was analyzed by high performance liquid chromatography (HPLC), as described by Elisia and Kitts [24]. Breast milk samples were thawed, mixed with 1 mL of 100 mM DL-dithiothreitol by mechanical inversion for 30 s in amber microcentrifuge tubes, and treated with 300 μL of 0.56% metaphosphoric acid. The mixture was shaken for 30 s, centrifuged at 10 °C, 10,000 rpm, and supernatants were filtered prior to injection into the HPLC system consisting of a Zorbax RX-C18 5 μL column (250 × 4.6 mm) at 45 °C and a mobile phase of 0.1 M NaH_2_PO_4_ (pH 2.5) buffer at 1.5 mL/min with detection at 254 nm. Alpha-tocopherol and beta-carotene were analyzed by HPLC, as previously described. Thawed breast milk (500 μL) was diluted with 500 μL of water followed by the addition of 500 μL of ethanol to precipitate breast milk proteins. The resulting mixture was extracted twice with 2 mL of hexane and extracts were evaporated under a stream of nitrogen. The residue was then reconstituted in 100 μL of methanol, and the sample filtered through a 0.2 μm nylon membrane into an amber HPLC vial for injection. The separation of alpha-tocopherol and beta-carotene was performed using a 3 μm Sphereclone column (150 mm × 4.6 mm) with ethanol as the mobile phase (1 mL/min). Alpha-tocopherol and beta-carotene were identified using fluorescence detection excitation with wavelengths of 292 and 450 nm, respectively.

### 2.5. Maternal Plasma

Blood samples were collected from GD participants only, in the arm median cubital vein using a peripheral venous catheter by a Registered Nurse (RN). Blood samples were collected at 29 weeks gestation (baseline; visit 1), 35 weeks gestation (visit 2), and again at 6–8 weeks postpartum (visit 3). Blood samples, collected at 29 and 35 weeks, corresponded with lactogenesis stage I [33].

Plasma samples collected in spray-coat K2 EDTA tubes were spun at 2500 rpm at 4 °C for 15 min using a centrifuge, transferred into 2 mL amber microtubes, and stored at −80 °C. Plasma samples were transported on dry ice in amber microtubes wrapped in aluminum foil to the Guelph Research and Development Centre, Agriculture and Agri-Food Canada, Guelph, Ontario. Antioxidant capacity was analyzed by ORAC measured in Trolox Equivalents (mM TE) in quadruplicate. ORAC was compared between participants in the treatment standard care and low glycemic index groups. Additionally, all plasma analyses included a standard internal control for plates. Plasma samples were analyzed at the Guelph Research and Development Centre, Agriculture and Agri-Food Canada, (Guelph, ON, Canada).

### 2.6. Maternal Dietary Intake

The maternal dietary intake of all foods, beverages, and vitamin and mineral supplements consumed was recorded using a three-day food record from women in both the GD and NG groups, administered by a trained Registered Dietitian or Dietetic Intern. Maternal dietary intake records were collected at 29 weeks gestation, 35 weeks gestation, and 6–8 weeks postpartum. Dietary intake was analyzed by the ESHA Food Processor Nutrition Analysis software version 10.15x (ESHA Research, Salem, OR, USA) for energy and macronutrients (carbohydrates, fat, and protein, and dietary antioxidants, including alpha-tocopherol, beta-carotene, and ascorbic acid).

### 2.7. Statistical Analysis

Mean and SDs were calculated for participants’ age, body mass index (BMI; pre- and post-pregnancy), and time of breast milk collection. Counts and percentages were calculated for demographics (including history of GD and ethnicity). Statistical analyses were conducted using GraphPad Prism version 6.0c (San Diego, CA, USA). The criterion for statistical significance was 2-tailed *p* < 0.05.

#### 2.7.1. Breast Milk

The breast milk data were not normally distributed according to the Kolmogorov–Smirnov test, and therefore, data were Log2 transformed. Student’s *t*-test was used to compare group means of ORAC, alpha-tocopherol, beta-carotene, and ascorbic acid concentrations in breast milk from women diagnosed with and without GD, respectively, at 8 weeks postpartum. A regression analysis was carried out to examine the relationship between breast milk antioxidant concentrations and breast milk ORAC, and maternal plasma ORAC and breast milk ORAC.

#### 2.7.2. Maternal Plasma

Student’s *t*-test was used to determine significant differences between plasma ORAC and breast milk ORAC, alpha-tocopherol, beta-carotene, and ascorbic acid concentrations in women diagnosed with and without GD. A paired *t*-test was used to determine changes in ORAC between participants in the GD group at baseline and 35 weeks gestation, and baseline and six to eight weeks postpartum.

#### 2.7.3. Maternal Dietary Intake

Pearson correlations were performed to determine the relationships among concentrations of each of the antioxidant vitamins measured in breast milk and breast milk ORAC, breast milk antioxidant vitamin concentrations, with dietary antioxidants at 6 to 8 weeks postpartum, and antioxidant vitamin concentrations in breast milk. No adjustments were undertaken for multiple comparisons. A regression analysis was carried out to examine the relationship between maternal dietary and breast milk antioxidant concentrations.

## 3. Results

**Figure 1:**
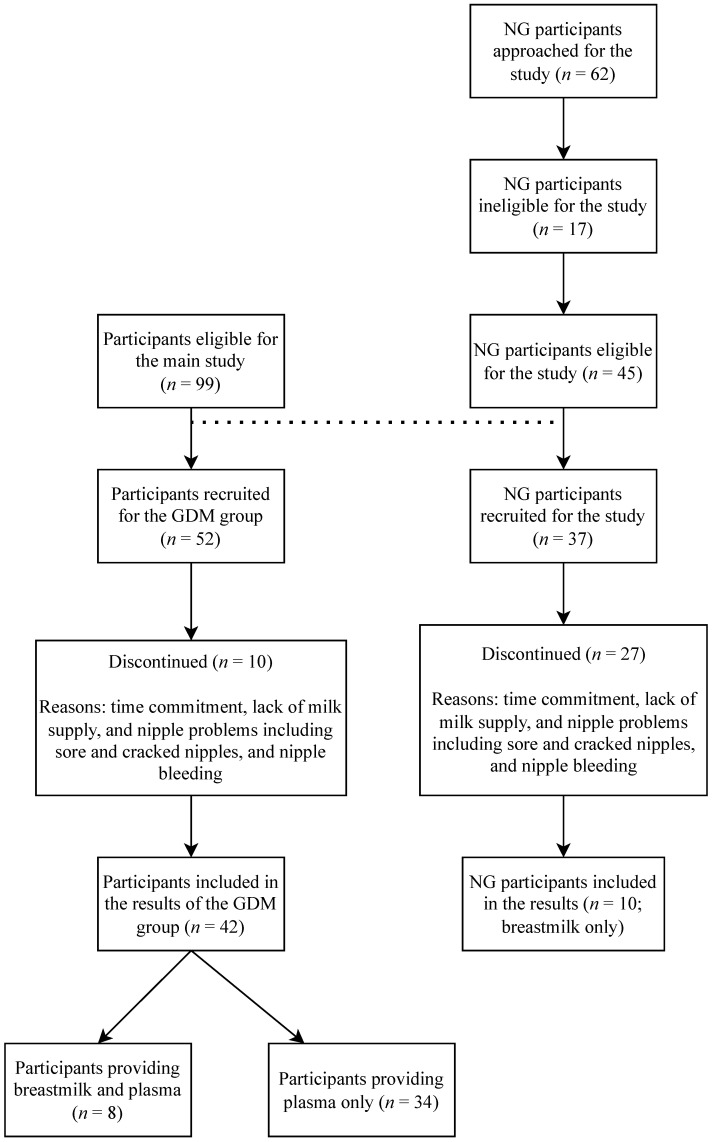
Recruitment CONSORT diagram. The dotted line represents the relationship between the GDM and NG groups. GDM, Gestational Diabetes Mellitus; NG, Normoglycemic.

**Figure 2:**
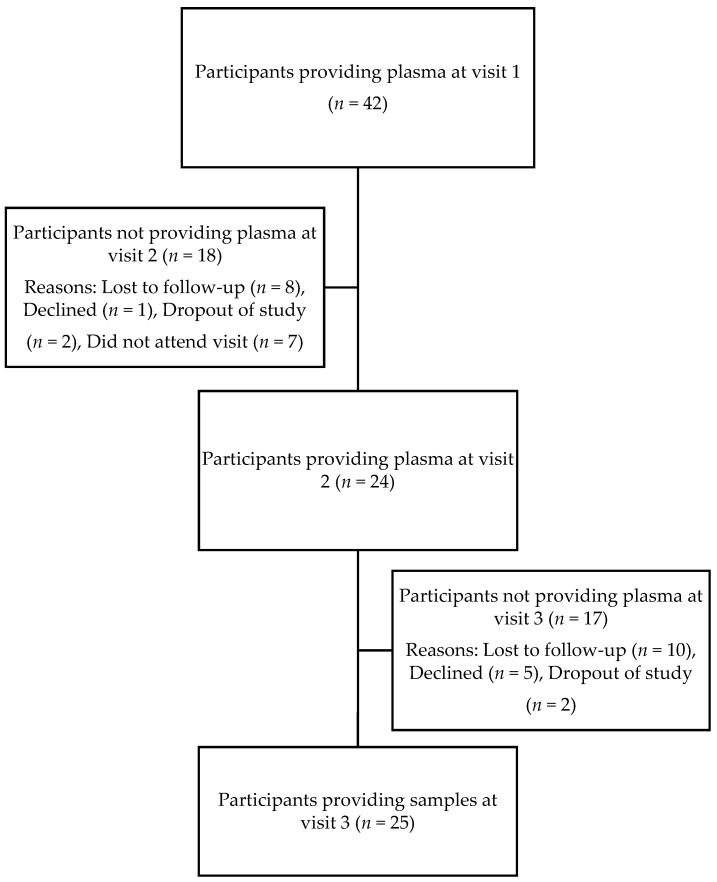
Sample size (counts) attrition in the context of plasma collection.

In this study, of the 52 pregnant women diagnosed with GD, 42 were included in this analysis, with 8 GD participants providing both breast milk and plasma samples and 34 participants with GD providing plasma samples only. The ten withdrawals from the GD group were due to reasons including milk supply, soreness of nipples, and time commitments. Of the thirty-seven NG pregnant women recruited, 10 were included in the analysis. Twenty-seven participants withdrew from the study due to reasons similar to the participants in the GD group (lack of milk supply, nipple difficulties, and time commitments). GD participants had the opportunity to provide both breast milk and blood samples, while the NG participants only provided breast milk samples (Figure 1). The attrition for plasma collection can be found in Figure 2.

The characteristics of the 18 (8 GD; 10 NG) participants that provided breast milk samples are presented in Table 1. The 10 NG participants had a mean age of 31.9 ± 2.6 years, a mean pre-pregnancy body mass index (BMI) of 26.0 ± 8.7 kg/m^2^, a mean postpartum BMI of 28.4 ± 8.1 kg/m^2^, and the majority were European (60% total). The 8 GD participants had a mean age of 33.9 ± 3.1 years, a mean pre-pregnancy BMI of 26.4 ± 3.5 kg/m^2^, a mean postpartum BMI of 26.8 ± 3.1 kg/m^2^, and were of various ethnicities. One (13% total) of the GD participants had a history of GD and two (25% total) had a first degree relative with GD, while none of the NG participants had a history of GD and three (30% total) had a first degree relative with GD (Table 1).

The dietary intakes of macronutrients and antioxidants from the two groups of women are presented in Table 2. Women with GD ingested significantly fewer calories compared to NG women (GD = 1810 kcal vs. NG = 2220 kcal; *p* = 0.032), and alpha-tocopherol levels were higher (GD = 10.49 mg vs. NG = 4.26 mg; *p* = 0.009). However, there were no statistically significant differences between groups for carbohydrates (GD = 228 g vs. NG = 284 g; *p* = 0.090), fibre (GD = 19.7 g vs. NG = 24.3 g; *p* = 0.19), fat (GD = 61.3 g vs. NG = 78.8 g; *p* = 0.45), protein (GD = 103.2 g vs. NG = 86.0 g; *p* = 0.60), beta-carotene (GD = 3950 µg vs. NG = 2960 µg; *p* = 0.92), or ascorbic acid (GD = 130 mg vs. NG = 130 mg; *p* = 0.15) (Table 2).

Plasma ORAC values at 29 weeks gestation had a mean value (±SEM) of 14,395 ± 232 mM TE and increased to a mean value (± SEM) of 15,989 ± 398 mM TE at 35 weeks gestation (*p* = 0.006). Between 29 weeks gestation and 35 weeks gestation, the net change in mean plasma ORAC (± SEM) was significantly increased by 734 ± 368 mM TE (*p* = 0.006). Additionally, plasma ORAC values at 6 weeks postpartum (15989 ± 398 mM TE) had a significant net change, increasing by 1753 ± 334 mM TE (*p* = 0.006).

The results of the antioxidant capacity (ORAC) and dietary antioxidant concentrations in mothers’ breast milk are presented in Table 3. There were no statistically significant differences in the median ORAC (GD = 2.33 mmol/L vs. NG = 1.83 mmol/L; *p* = 0.06), beta-carotene (GD = 20.9 nmol/L vs. NG 43.9 nmol/L; *p* = 0.12), alpha-tocopherol (GD = 3.92 μmol/L vs. NG = 4.57 μmol/L; *p* = 0.33), and ascorbic acid (GD = 344 μmol/L vs. NG = 391 μmol/L; *p* = 0.35) concentrations in the breast milk between women with GD versus NG women.

**Figure 3:**
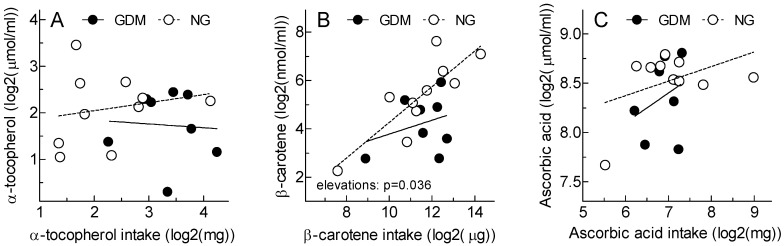
Dietary antioxidant vitamins: intakes versus breast milk concentrations. Solid line: GD; Dotted line: NG. Breast milk concentrations (Y-axis) plotted on intakes (x-axis) of alpha-tocopherol (**A**), beta-carotene (**B**), and ascorbic acid (**C**) in 8 women with gestational diabetes mellitus (GDM) and 10 women without gestational diabetes (NG). Correlation coefficients (*p*-values) in GDM vs. NG, respectively: panel A, *r* = 0.065 (ns) vs. *r* = 0.194 (ns); panel B, *r* = 0.310 (ns) vs. *r* = 0.827 (*p* = 0.0025); panel C, *r* = 0.317 (ns) vs. *r* = 0.429 (ns). Panel B: the regression line for GDM has a significantly lower elevation that that for NG (*p* = 0.036).

Figure 3 illustrates the relationship between antioxidant vitamin intakes and breast milk concentrations. There were no significant relationships between dietary alpha-tocopherol (panel A) and ascorbic acid (panel C) intake and breast milk concentrations in either the GD or NG groups. Additionally, there was no significant relationship between dietary beta-carotene (panel B) intake and breast milk concentrations for the GD group. However, a significant relationship was observed for the NG group (*p* = 0.0025). Moreover, the elevation (slope) of the beta-carotene regression line for the GD group was significantly lower than the regression line for the NG group (*p* = 0.036).

**Figure 4:**
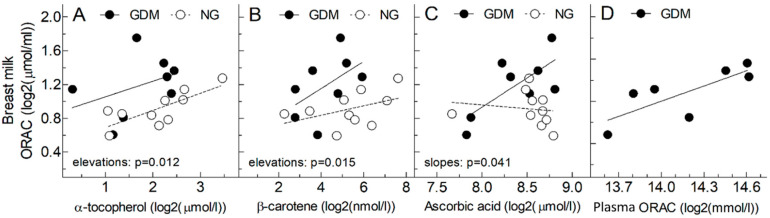
Breast milk antioxidant capacity versus antioxidant vitamin concentrations and plasma ORAC. Solid line: GD; Dotted line: NG. Breast milk ORAC plotted on breast milk antioxidant concentrations of alpha tocopherol (**A**), beta-carotene (**B**), and ascorbic acid (**C**), and plasma ORAC (**D**) in 8 women with gestational diabetes mellitus (GDM).

The relationship between breast milk ORAC and the antioxidant concentrations for alpha-tocopherol, beta-carotene, and ascorbic acid, along with the relationship between breast milk and plasma ORAC in GD mothers, is presented in Figure 4, panels A–D, respectively. There was no significant correlation between breast milk ORAC and alpha tocopherol concentrations (panel A) for the GD group; however, there was a significant correlation for the NG group (*p* = 0.010). No significant correlations were found between breast milk ORAC and beta-carotene (panel B) for either the GD or the NG group. However, there was a significant correlation between breast milk ORAC and ascorbic acid concentrations (panel C) for the GD group (*p* = 0.043), but not in the NG group. A significant correlation was found between breast milk ORAC and plasma ORAC (panel D; *p* = 0.039). In addition, the elevations of the alpha-tocopherol and beta-carotene GD regression line were significantly higher than the NG regression line: *p* = 0.012 and *p* = 0.015, respectively. Furthermore, the slope of the GD regression line of ascorbic acid was significantly higher than the NG regression line (*p* = 0.041).

## 4. Discussion

It was hypothesized that women diagnosed with GD would have lower antioxidant concentrations, in both plasma and breast milk, than women without GD; however, no significant differences were observed between groups in this study. The secondary data analysis compared mothers’ breast milk and plasma antioxidant capacities, using ORAC and direct measures of alpha-tocopherol, beta-carotene, and ascorbic acid milk concentrations in women diagnosed with and without GD during their most recent pregnancy. Maternal dietary intake of alpha-tocopherol and ascorbic acid was not significantly related to alpha-tocopherol and ascorbic acid breast milk concentrations in women with and without GD. However, beta-carotene maternal intake was significantly related to beta-carotene breast milk concentrations in women without GD, which is in agreement with previous research [34,35,36].

This study detected a significant correlation between beta-carotene intake and beta-carotene concentrations in breast milk in the NG group, but not in the GD group. Dietary intake of beta-carotene is known to directly affect beta-carotene concentrations in breast milk; however, other factors affecting beta-carotene concentrations include the fat content of breast milk, the time of breast milk collection, and the duration of breastfeeding [34]. Typically, beta-carotene concentrations in breast milk are approximately 49.4 nmol/L for women without GD [34,35]; in the current study, the median beta-carotene concentrations for the GD and NG groups were 20.9 nmol/L and 43.9 nmol/L, respectively. Therefore, in the future, factors known to affect beta-carotene concentration should be accounted for, such as time of breast milk collection. Although not statistically different, further exploration of our hypothesis is warranted [34].

The current study observed no significant associations between alpha-tocopherol concentrations in breast milk for both the GD and NG groups, with medians of 3.92 μmol/L and 4.57 μmol/L. Additionally, no correlations were found between alpha-tocopherol breast milk concentrations and dietary intake, despite GD participants’ dietary intakes of alpha-tocopherol being nearly four times that of NG participants. This is in agreement with previous studies on alpha-tocopherol concentrations in breast milk, which have indicated that dietary intake of alpha-tocopherol (or vitamin E) does not affect concentrations because fat soluble vitamins can be mobilized from maternal fat stores [37,38].

Ascorbic acid breast milk concentrations observed in both GD, and NG participants were not significant. The non-significant differences in ascorbic acid concentrations could be explained as an ascorbic acid regulatory mechanism, in which mammary tissues control breast milk antioxidant concentrations [39]. Byerley and Kirksey [40] determined that, when well-nourished lactating women without GD increased their intake of ascorbic acid over ten-fold, there was no effect on breast milk ascorbic acid concentrations. Although breast milk ascorbic acid concentrations were not affected, a significant increase in urinary ascorbic acid excretions were reported [40]. Their observation suggests a regulatory mechanism for the mammary utilization of ascorbic acid. In the future, outcomes including ascorbic acid excretions in urinary output should be compared to both ascorbic acid breast milk concentrations and plasma concentrations.

Breast milk ORAC was related to breast milk alpha-tocopherol and beta-carotene concentrations in NG and GD participants, but the only significant correlation observed was between ORAC and alpha-tocopherol in NG participants. In addition, breast milk ORAC was positively correlated with breast milk ascorbic acid in GD but not NG, with the slope of the regression line being significantly greater in GD than NG. A larger number of participants will be required to confirm this finding.

Breast milk ORAC in women with GD was positively correlated with plasma ORAC. Additionally, plasma ORAC was significantly increased between 29 weeks and 35 weeks gestation women with GD, and between 29 weeks gestation and 8 weeks postpartum [41]. Research is very limited surrounding plasma ORAC in women with GD during pregnancy and postpartum. A recent study by Zygula et al. [42] measured oxidative stress markers in plasma between women with and without GD; the women with GD were either treated by diet or by insulin. Therefore, women with GD who were treated with diet had significantly higher plasma ORAC compared to women treated with insulin and women without GD [42]. The researchers hypothesize that the increased antioxidant capacity in the diet-controlled GD group may be because the group had a more balanced diet compared to the insulin-controlled and NG group, potentially due to the fear of hyperglycemia or hypoglycemia [42]. More research is needed to determine the effects of diet on both plasma ORAC and breast milk ORAC.

The value of antioxidant richness in breast milk is conceivably important to protect nursing infants against OS [17,18,19,20,21]. Due to the limitations of recruitment and retention of subjects in this study, the sample size is relatively small, and hence our findings at this time are regarded as preliminary. From this work, it has been estimated that to establish 80% power to detect a significant difference in ORAC, a study such as this would require 22 NG women and 22 women with GD.

Another challenge of this experiment was that, for most women diagnosed with GD, blood glucose will return to normal after their infant is born [43] and therefore a portion of participants with GD may have been experiencing normoglycemia at the time of breast milk sample collection, which equates with the final blood collection (visit 3) in our study. The large number of dropouts in our study has also been reported in other studies with similar research objectives and samples, and it is known that factors such as low milk supply, nipple issues, and lack of free time once the baby was born are constraints to retain subjects. Previous research has found that many women fear having low milk supply [44], which could lead to a reluctance to participate where breast milk collection is required. Previous studies have identified solutions to the barriers of participation in studies involving breast milk collection; these include home visits, childcare, and lactation support [45]. Despite observing differences in the health status of mothers (GD vs. NG), the antioxidant capacity of breast milk remains somewhat comparable, suggesting that GD may not impact the quality of breast milk that the infants receive.

## 5. Conclusions

The findings from this secondary data analysis provide information for hypotheses development. Although there were no significant statistical differences detected between GD and NG participants, this analysis provides insights relevant to ongoing clinical assessment and the metabolic understanding of the relationship between maternal diet, blood, and breast milk. Although these data indicate that the antioxidant capacity of breast milk may not be associated with GD or postnatal dietary antioxidant intake, plasma and breast milk ORAC were correlated, highlighting an assay that could be useful in assessing the relationships between maternal diet, blood, and breast milk, and infant plasma. This work reiterates the relevance of measuring individual antioxidants to gain a better understanding of mechanisms, given the differences observed between lipophilic and hydrophilic nutrient antioxidants.

## Figures and Tables

**Table 1:** Sample characteristics.

Characteristics	GD (*n* = 8)	NG (*n* = 10)	*p*-Value
Age (years) *	33.9 ± 3.1	31.9 ± 2.6	ns
Pre-pregnancy BMI (kg/m^2^) *	26.4 ± 3.5	26.0 ± 8.7	ns
Postpartum BMI (kg/m^2^) *	26.8 ± 3.1	28.4 ± 8.1	ns
Time of breast milk collection (week postpartum) *	8.3 ± 1.9	7.3 ± 1.3	ns
GD history	13%	0%	ns
First degree relative with GD	25%	30%	ns
Ethnicity (%)			ns
Aboriginal	0%	10%	
Caucasian	37.5%	60%	
East Asian	25%	-	
Indian	-	10%	
Latin American	12.5%	-	
Middle Eastern	-	20%	
Southeast Asian	12.5%	-	
Other	12.5%	-	

* Values represent means ± SD; BMI, Body mass index; GD, Gestational diabetes.

**Table 2:** Maternal dietary intakes.

	GD (*n* = 8)	NG (*n* = 10)	*p*-Value
Energy (kcal) Intake	1810 (1710, 2170)	2220 (2180, 2380)	0.032
Carbohydrates (g)	228 (207, 259)	284 (243, 308)	0.090
Fiber (g)	19.7 (15.3, 27.7)	24.3 (18.3, 32.3)	0.19
Fat (g)	61.3 (49.0, 79.9)	78.8 (72.0, 105.4)	0.45
Protein (g)	103.2 (82.2, 108.0)	86.0 (72.8, 107.8)	0.60
Beta-carotene (µg)	3950 (2520, 5240)	2960 (1920, 5590)	0.92
Alpha-tocopherol (mg)	10.49 (8.10, 13.24)	4.26 (3.22, 6.75)	0.009
Ascorbic acid (mg)	130 (105, 147)	130 (100, 152)	0.15

Values are medians (interquartile range). *p*-values are by unpaired *t*-test on log-transformed values; GD, Gestational diabetes; NG, Normoglycemic.

**Table 3:** Antioxidant capacity and vitamin content of breast milk.

	GD (*n* = 8)	NG (*n* = 10)	*p*-Value
ORAC (mmol/L)	2.33 (2.03, 2.62)	1.83 (1.74, 2.02)	0.06
Beta-carotene (nmol/L)	20.9 (10.8, 31.5)	43.9 (28.4, 77.4)	0.12
Alpha-tocopherol (μmol/L)	3.92 (2.51, 4.99)	4.57 (2.89, 5.9)	0.33
Ascorbic acid (μmol/L)	344 (283, 404)	391 (368, 408)	0.35

Values are medians (interquartile range). *p*-values are by unpaired *t*-test on log-transformed values; ORAC, Oxygen radical absorbance capacity; NG, Normoglycemic; GD, Gestational diabetes.

## Data Availability

The data presented in this study are available in the article.
